# Amygdala Volume is Associated with ADHD Risk and Severity Beyond Comorbidities in Adolescents: Clinical Testing of Brain Chart Reference Standards

**DOI:** 10.1007/s10802-024-01190-0

**Published:** 2024-03-14

**Authors:** Ádám Nárai, Petra Hermann, Alexandra Rádosi, Pál Vakli, Béla Weiss, János M. Réthelyi, Nóra Bunford, Zoltán Vidnyánszky

**Affiliations:** 1 Brain Imaging Centre, HUN-REN Research Centre for Natural Sciences, Budapest, Hungary; 2 Doctoral School of Biology and Sportbiology, Institute of Biology, Faculty of Sciences, University of Pécs, Pécs, Hungary; 3 Clinical and Developmental Neuropsychology Research Group, Institute of Cognitive Neuroscience and Psychology, HUN-REN Research Centre for Natural Sciences, Budapest, Hungary; 4 Department of Psychiatry and Psychotherapy, Faculty of Medicine, Semmelweis University, Budapest, Hungary; 5 Doctoral School of Mental Health Sciences, Semmelweis University, Budapest, Hungary

**Keywords:** Brain Chart, Normative Modeling, ADHD, Adolescent, MRI

## Abstract

Understanding atypicalities in ADHD brain correlates is a step towards better understanding ADHD etiology. Efforts to map atypicalities at the level of brain structure have been hindered by the absence of normative reference standards. Recent publication of brain charts allows for assessment of individual variation relative to age- and sex-adjusted reference standards and thus estimation not only of case-control differences but also of intraindividual prediction. *Methods.* Aim was to examine, whether brain charts can be applied in a sample of adolescents (*N* = 140, 38% female) to determine whether atypical brain subcortical and total volumes are associated with ADHD at-risk status and severity of parent-rated symptoms, accounting for self-rated anxiety and depression, and parent-rated oppositional defiant disorder (ODD) as well as motion. *Results.* Smaller bilateral amygdala volume was associated with ADHD at-risk status, beyond effects of comorbidities and motion, and smaller bilateral amygdala volume was associated with inattention and hyperactivity/impulsivity, beyond effects of comorbidities except for ODD symptoms, and motion. *Conclusions.* Individual differences in amygdala volume meaningfully add to estimating ADHD risk and severity. Conceptually, amygdalar involvement is consistent with behavioral and functional imaging data on atypical reinforcement sensitivity as a marker of ADHD-related risk. Methodologically, results show that brain chart reference standards can be applied to address clinically informative, focused and specific questions.

**Supplementary Information:**

The online version contains supplementary material available at 10.1007/s10802-024-01190-0.

## Introduction

Attention-deficit/hyperactivity disorder (ADHD), characterized by developmentally-inappropriate inattention, hyperactivity and/or impulsivity as well as functional impairment (American Psychiatric Association, 2022), is an early-onset yet often lifelong, prevalent, and costly disorder. ADHD is typically diagnosed in the early school years (American Psychiatric Association, 2022) and its symptoms persist, in 50% of cases, at a diagnostic (Roy et al., 2016), and in 65% of cases, at an impairing (Faraone et al., 2006) level into adulthood. Worldwide, ADHD is diagnosed in ~ 5–9% of children and adolescents – hundreds of millions of youth (Danielson et al., 2018; Salari et al., 2023; Thomas et al., 2015) and ADHD-associated annual costs amount to hundreds of billions of dollars (Sciberras et al., 2022). Attenuating the personal and societal burden of ADHD necessitates improvement of early identification of at-risk individuals and of personalized prevention, which in turn necessitates improvements in understanding ADHD etiology.

Alterations or atypicalities in brain function and structure are at the center of conceptual frameworks explaining ADHD etiology (Barkley, 1997; Castellanos et al., 2006; Castellanos & Tannock, 2002; Halperin & Schulz, 2006; Sonuga-Barke, 2003; Sonuga-Barke et al., 2010) and ample data indicate differences between children and adults with and without ADHD in brain volume. Specifically, meta-analytic findings pooling region of interest brain volume (Valera et al., 2007) and voxel-based morphometry (VBM) (Ellison-Wright et al., 2008; Frodl & Skokauskas, 2012; Nakao et al., 2011; Norman et al., 2016) studies consistently indicate reduced volumes in individuals with ADHD in certain parts of the basal ganglia (caudate, putamen, globus pallidus). Albeit less consistently, results also suggest reduced volumes in the cerebrum, corpus callosum (splenium), insula, medial prefrontal cortex, ventromedial orbitofrontal cortex, and anterior cingulate cortex (Ellison-Wright et al., 2008; Frodl & Skokauskas, 2012; Nakao et al., 2011; Norman et al., 2016; Valera et al., 2007). More recently, larger samples allowed for the detection of case-control effect sizes observed in other psychiatric disorders and data showed reduced volumes in ADHD in regions beyond those observed previously, including in the accumbens, amygdala, hippocampus and intracranial volume (ICV) (Hoogman et al., 2017).

Indicative of clinical utility, altered brain volume, in turn, is associated with ADHD clinical features in clinical and in the general populations. Greater severity of symptoms is associated in clinical samples with smaller volumes of the caudate, cerebellum, and frontal and temporal gray matter (Castellanos et al., 2002) and in general population samples with smaller total volume (Hoogman et al., 2012). Both severity of symptoms and the ADHD syndrome are also associated in community samples with slower cortical thinning predominantly in prefrontal cortical regions, bilaterally in the middle frontal/premotor gyri, extending down the medial prefrontal wall to the anterior cingulate; the orbitofrontal cortex; and the right inferior frontal gyrus (Shaw et al., 2011).

Yet, the majority of available data on structural brain differences in children and adults with ADHD have been obtained in case-control designs. Case-control designs, although informative about the extent to which - *at the group level -* individuals with ADHD differ from individuals without ADHD, are less informative about the extent to which *any given individual with ADHD* is atypical. An emerging but robust body of work indicates the need to at least augment if not shift focus from between-groups comparisons to the assessment of differences at the individual level. Specifically, data show considerable within-group heterogeneity both in ADHD and in the general population (Braver et al., 2010; Buss, 1991; Fair et al., 2012) and thus suggest that personalization of behavioral medicine necessitates estimation and knowledge of differences at the individual level.

In case of anthropometric traits such as head circumference, height and weight, individual differences can be quantified against reference standards (i.e., normative growth charts). As disorders like ADHD, autism spectrum disorder (ASD), or schizophrenia are caused/ characterized by atypical brain development, the ability to quantify individual differences is at least comparably if not especially relevant in case of clinical neuroimaging. Yet, until most recently (Bethlehem et al., 2022; Rutherford et al., 2022), no brain development reference standards were available. Capitalizing on advances across neuroimaging and statistical techniques and on availability of large datasets, brain charts have been generated to define reference standards for structural brain measures including global features such as total cortical and subcortical gray matter volume, total white matter volume, and total ventricular cerebrospinal fluid (Bethlehem et al., 2022) as well as specific estimates for 188 different brain regions (Rutherford et al., 2022) across ages and sexes (Bethlehem et al., 2022; Rutherford et al., 2022). Data obtained for the development and validation of brain charts indicate the charts are appropriate for dissecting biological heterogeneity in clinical disorders (e.g., anxiety and depression; ADHD and ASD; and degenerative disorders) both in terms of global features (Bethlehem et al., 2022) and in summary metrics (representing deviation patterns across individual brain regions) (Rutherford et al., 2022) of brain development.

### Current Study

As a next step towards demonstrating clinical potential of brain charts, the aim in the current study was to examine whether brain development reference standards can be applied for addressing specific questions in estimating clinical effects in a deeply phenotyped sample. Specifically, aim was to examine whether brain charts can be applied in a sample of adolescents to determine whether atypical brain region volumes predict ADHD at-risk status and severity of inattention (IA) and hyperactivity/impulsivity (H/I), accounting for the effects of symptoms of common comorbidities including anxiety, depression, and oppositional defiant disorder (ODD) as well as the effects of motion.

In addition to estimating effects of total cranial volume, we also modeled effects of individual subcortical regions. We elected to focus on subcortical rather than cortical regions for the following reasons. Available data are inconsistent with a key role of the prefrontal cortex (PFC) (and its interconnections with the striatum and other subcortical structures) in the causing of ADHD. First, in contrast to adults, most children with early frontal lobe damage do not exhibit ADHD symptoms and second, the normative developmental trajectory and emergence of ADHD is inconsistent with the ontogenetic development of the PFC or the executive functions it mediates (for review, see Halperin & Schulz, 2006). Conversely, data are consistent with a role of the PFC in the manifestation and remission of ADHD. The developmental trajectory of the PFC and the executive functions it mediates closely parallel the apparent attenuation of ADHD severity in adolescence and young adulthood (for review, see Halperin & Schulz, 2006). Further support for our focus on subcortical regions is provided by findings on noncortical neural dysfunction in causing ADHD. First, such dysfunction is present early in ontogeny. For example, early, pre- and perinatal brain insults alter norepinephrine metabolism in the hindbrain and dopamine innervation of the striatum, and the cerebellum is among the most vulnerable regions to early insult. These regions and the functions they mediate – including alertness and arousal (hindbrain); reinforcement and reward processing (striatum); and attention and temporal processing (cerebellum) are consistently implicated in ADHD, making them candidates for a key role in the cause of ADHD (for review, see Halperin & Schulz, 2006). Second, these dysfunctions remain relatively static throughout the lifetime (but become compensated in adolescence and young adulthood by “top-down” regulatory or executive control) and third, they are not associated with the attenuation of ADHD severity across development (for review, see Halperin & Schulz, 2006). In combination with these conceptual considerations as well as meta-analytic data consistently indicating reduced subcortical volumes in ADHD (Ellison-Wright et al., 2008; Frodl & Skokauskas, 2012; Nakao et al., 2011; Norman et al., 2016) but less consistently indicating reduced cortical volumes in ADHD (Ellison-Wright et al., 2008; Frodl & Skokauskas, 2012; Nakao et al., 2011; Norman et al., 2016; Valera et al., 2007), our focus herein is on modeling effects of individual subcortical regions.

Psychiatric comorbidity in ADHD is less the exception and more the rule: common comorbidities in ADHD observed across clinical and community samples include anxiety disorders, depression, and ODD, with comorbidity rates around 25% for anxiety (D’Agati et al., 2019), 12–50% for depression (Gnanavel et al., 2019), and 30–50% for ODD (Gnanavel et al., 2019). To avoid introduction of confounds, comorbidities were accounted for in analyses.

As ADHD diagnostic criteria and corresponding interview and scale measures indicate ADHD is associated with locomotor hyperactivity (though behavioral data are mixed (Castellanos & Tannock, 2002), ADHD may be associated with greater motion in the MRI (Gilmore et al., 2021; Pardoe et al., 2016) and this may confound measures of brain anatomy (Alexander-Bloch et al., 2016; Reuter et al., 2015). Thus, effects of motion were also accounted for in analyses.

Analyses were replicated, as in (Rutherford et al., 2023), using the output of traditional MRI preprocessing methods (hereafter: raw data) to compare findings obtained using normative modeling with those obtained not applying reference standards.

## Methods

### General Procedure

Data analyzed in the current study were obtained during the first three assessment sessions of the second (baseline, i.e., T1) year of a larger longitudinal project, the Budapest Longitudinal Study of ADHD and Externalizing Disorders (BLADS) study. Exclusionary criteria were cognitive ability ≤ the percentile rank corresponding to a full-scale IQ score of 80 (Wechsler, 2003, 2008); meeting diagnostic criteria for autism spectrum (severity ≥ 2), bipolar, obsessive-compulsive, or psychotic disorder on the Structured Clinical Interview for DSM-5 Disorders, Clinical Version (SCID-5 CV) (First et al., 2016); neurological illness; and visual impairment (uncorrected, impaired vision < 50 cm).

Parents and participants provided written informed consent (and assent) and then participants underwent a series of tests, including assessment of cognitive ability and a clinical interview, followed by genetic sampling and questionnaires (first assessment session) and an EEG measurement and questionnaires (second assessment session), and an MRI measurement (third assessment session). Parents completed questionnaires using the Psytoolkit platform (Stoet, 2010, 2017) and the Qualtrics software, Version June 2020–March 2021 (Qualtrics, Provo, UT). This research was approved by the National Institute of Pharmacy and Nutrition (OGYÉI/27,030/2020) and has been performed in accordance with the ethical standards laid down in the 1964 Declaration of Helsinki and its later amendments.

ADHD classification was determined using parent-report on the ADHD Rating Scale-5 (ARS-5) (DuPaul et al., 2016). To be classified as at-risk for ADHD, adolescents had to meet a total of ≥ 4 of the Diagnostic and Statistical Manual of Mental Disorders (5th ed.; DSM-5) ADHD symptoms (from either the IA or the H/I domain).

### Participants

Participants were adolescents from the larger community sample of adolescents oversampled for ADHD who participated in the MRI measurement. The analysis sample for this study was comprised of *N* = 140 adolescents.

### Measures

#### Adolescent Self-Report Measures

##### Anxiety Problems and Depressive Problems

The Youth Self-Report (YSR) (Achenbach & Rescorla, 2001) is a 112-item self-report questionnaire for adolescents (ages 11–18) assessing aspects of adaptive and impaired functioning. The YSR measures adaptive functioning through *competence scales*: academic performance, activities, and social competence and impaired functioning via *Diagnostic and Statistical Manual of Mental Disorders (DSM)-oriented scales*: anxiety problems, depressive problems, somatic problems, attention-deficit/ hyperactivity problems, oppositional defiant problems, and conduct problems; as well as *syndrome scales*: anxious/depressed, depressed/withdrawn, somatic complaints, attention problems, social problems, thought problems, aggressive behavior, rule-breaking behavior, externalizing problems and internalizing problems. Respondents rate items on a 3-point scale (0 – ‘Not True’, 1 – ‘Somewhat or Sometimes True’, 2 – ‘Very True or often True’).

Prior findings indicate both the original (Achenbach & Rescorla, 2001) and the Hungarian translation of the YSR (Rádosi et al., 2023) have acceptable psychometric properties.

In the current study, the anxiety and the depressive problems subscales were used in analyses.

#### Parent-report Measures

##### ADHD Risk and Severity

The ADHD Rating Scale-5 (ARS-5) (DuPaul et al., 2016) is a 30-item parent- and teacher-report measure of the past 6-month presence and severity of DSM-5 ADHD symptoms (9 inattentive symptom items and 9 hyperactivity/impulsivity symptom items) and functional impairment across six domains: relationship with significant others (family members for the home version), relationship with peers, academic functioning, behavioral functioning, homework performance and self-esteem (2 × 6 impairment items, with one set corresponding to inattention and one to hyperactivity/impulsivity). Parents and teachers rate items on a four-point scale ranging in case of symptoms from 0 (never or rarely) to 4 (very often) and in case of impairment from 0 (no problem) to 3 (severe problem), with higher scores indicating more severe symptoms and impairment. The ARS-5 is comprised of two symptoms scales, Inattention and Hyperactivity-Impulsivity, and a Total Scale. The ARS-5 is suitable for ages 5–17 years, with separate forms for children (5–10 years) and adolescents (11–17 years) and age-appropriate and DSM-5 compatible descriptions of symptoms. In the current study, the adolescent home (i.e., parent-report) version was used.

Prior findings indicate both the original (DuPaul et al., 2016) and the Hungarian translation of the ARS-5 (Bunford et al., 2023; Hámori et al., 2023; Rádosi et al., 2023) has acceptable psychometric properties. In the current sample, the ARS-5 total (ω = 0.950), as well as the inattention (ω = 0.947) and hyperactivity/impulsivity (ω = 0.910) subscales exhibited acceptable internal consistency. In the current study, the ARS-5 was used for ADHD classification and the total score as well as the IA and H/I subscales were used in statistical analyses.

##### Oppositional Defiant Disorder

The Disruptive Behavior Disorders-Rating Scale (DBD-RS) (Pillow et al., 1998) is a 45-item parent- and teacher-report measure of the presence and severity of DSM-III-R ADHD symptoms (9 inattentive symptom items and 9 hyperactivity/impulsivity symptom items), oppositional defiant disorder (8 items), and conduct disorder symptoms (15 items). Parents and teachers rate items on a four-point scale ranging from 0 (not at all) to 3 (very much), with higher scores indicating more severe symptoms. In the current study, the parent-report form was used and the oppositional defiant disorder items were of interest. Because items reflect DSM-III-R symptom wording, items were modified to match DSM-5 symptom wording (American Psychiatric Association, 2022).

Prior findings indicate both the original (Van Eck et al., 2010) and the Hungarian translation of the DBD-RS (Rádosi et al., 2023) have acceptable psychometric properties. In the current sample, the ODD subscale exhibited ω = 0.916 internal consistency. In the current study, the ODD subscale was used in analyses.

#### MRI Data Acquisition and Preprocessing

Structural imaging was performed using a magnetization-prepared rapid gradient-echo (MPRAGE) scan with 2-fold in-plane GRAPPA acceleration on a Siemens Magnetom Prisma 3T MRI scanner with the standard Siemens 32-channel head coil using the following parameters: isotropic 1 mm^3^ spatial resolution, repetition time (TR) = 2300 ms, echo time (TE) = 3 ms, inversion time (TI) = 900 ms, flip angle (FA) = 9°, field of view (FOV) = 256✕256 mm.

Subcortical segmentation of T1-weighted structural images and estimation of morphometric statistics was performed using FreeSurfer 7.1.1 with the built-in probabilistic atlas (Fischl et al., 2020). A novel normative modeling framework (Rutherford et al., 2022) was used to calculate deviation *z*-scores for each subcortical volume, total subcortical gray matter volume and left- and right hemispheric mean cortical thickness. The deviation *z*-scores were averaged between hemispheres for the subcortical ROIs. For estimation we used the lifespan_57K_82sites model (Rutherford et al., 2022) and adapted it to our own site using an in-house structural MRI dataset. For this procedure we used the publicly available codes and documentation (https://github.com/predictive-clinical-neuroscience/braincharts) provided by Rutherford et al. We used our in-house dataset as the adaptation set and the current study dataset as the test set. Our in-house dataset consisted of T1-weighted images of *N* = 379 control (i.e. without history of neurological or psychiatric illness) participants (*n* = 230 female, *M*_age_=41.95, *SD* = 19.02, range: 18–80 years) collected across our studies conducted during recent years. Each scan was performed on the same scanner at our site, using the same MPRAGE protocol as for the analyzed dataset.

### Analytic Plan

Logistic and linear regression models with Elastic-Net regularization (both L1 and L2 penalty terms added) were used to determine whether and how atypical brain morphometry is associated with ADHD at-risk status and IA and H/I severity. As ADHD risk status was determined using ADHD severity, first, we modeled ADHD risk status and second, we followed-up significant models with more in-depth analyses of IA and H/I severity modeled separately. Hyperparameter tuning was performed using 5-fold cross validation with 20 l1_ratio values between 0 and 1 on an inverse logarithmic scale and 20 automatically selected values for regularization strength. For the logistic regression classifier, stratified cross validation and balanced class weighting were used. The best hyperparameter combination was selected using Area Under the Receiver Operating Characteristic Curve (AUC) scores for the logistic and coefficient of determination (*R*^2^) scores for the linear regression models. Models were refitted and evaluated on the whole dataset to calculate final coefficients and goodness of fit measures (AUC and *R*^2^ scores). Statistical evaluation of the results was performed using two-tailed permutation tests with 1000 random permutations and an alpha level of 0.05.

Modeling was performed on both deviation *z*-scores of normative brain morphometry and on raw morphometric estimates. Since deviation *z*-scores are inherently corrected for age and sex, age and sex were only included as additional covariates when using raw morphometric estimates. Global models included the left- and right hemispheric mean cortical thickness and the total volume of subcortical gray matter. Subcortical models included the volumes of 8 subcortical ROIs (Thalamus, Caudate, Putamen, Pallidum, Hippocampus, Amygdala, Accumbens, Ventral Diencephalon) averaged between hemispheres. All models included the intracranial volume (ICV) as control for the variance in total brain size. To control for the possible effects of participant motion, we included the Euler number (calculated from the FreeSurfer output) as covariate in all models, as it was shown to be highly correlated with manual ratings of motion artifacts (Rosen et al., 2018). Furthermore, based on findings of a recent study (Provins et al., 2023) performed on our T1-weighted motion controlled dataset (Nárai et al., 2022), SNR derived image quality metrics (IQMs) of MRIQC 0.16.1 (Esteban et al., 2017), namely SNR and SNRd for gray matter (GM), white matter (WM) and cerebrospinal fluid (CSF) were also included as motion covariates in all models. Anxiety, depression, and ODD scores were also controlled for. As ODD was highly correlated with dependent variables and thus could mask effects of other predictors, each model was also fitted without the ODD variable to obtain a more in-depth understanding of the observed results. All features were standardized to aid interpretability of coefficients and modeling was performed in Python (3.11.4) using the scikit-learn (1.3.0) (Pedregosa et al., 2011) package.

### Data Availability

The datasets generated and/or analyzed during the current study are available from the corresponding authors on reasonable request.

## Results

### Descriptive Results

In the analysis sample of *N* = 140 adolescents (*M*_age_=15.686, *SD* = 1.029; 38% female), families represented a slightly above-average socioeconomic background (average family net income was in the 500 001-700 000 HUF/month range, with average family net income in Hungary being 147 000 HUF/month) (Hungarian Central Statistical Office, n.d.). For additional descriptive results, see Table 1.

**Table 1:** Descriptive results for the analysis sample

Descriptive results for the analysis sample.					
at-risk for ADHD *n* = 59 (42%)			not at-risk for ADHD *n* = 81 (58%)		
age (*M, SD*)	sex (%female)	ODD (%meets diagnostic criteria)	age (*M, SD*)	sex (%female)	ODD (%meets diagnostic criteria)
15.541, 1.039	25	39	15.791, 1.015	47	14
Combined Sample					
ADHD research diagnosis^a^ *n* (%)	ODD research diagnosis^b^ *n* (%)	Anxiety Problems T score (*M, SD*)	Depression Problems T score (*M, SD*)	PRI percentile rank (*M, SD*)	VCI percentile rank (*M, SD*)
38 (27)	34 (24)	54.860, 6.423	55.74, 7.883	57, 25.196	70, 22.305

*Notes.*^a^= present ≥ 6 (youth < 17 years old) or 5 (youth ≥ 17 years old) IA or H/I symptoms and exhibit impairment (i.e., rating of ≥ 2 = moderate impairment) in at ≥ 3 areas of functioning on the ADHD Rating Scale-5-Home Version (ARS-5) (DuPaul et al., 2016); ^b^ = present ≥ 6 ODD symptoms on the Disruptive Behaviour Disorders Rating Scale (DBD-RS) (Pillow et al., 1998); PR = perceptual reasoning index; VCI = verbal comprehension index

Of adolescents at-risk for ADHD, *n* = 12 were currently prescribed ADHD pharmacological treatment and *n* = 2 were currently prescribed non-ADHD pharmacological treatment. Of those prescribed ADHD pharmacological treatment, 7 were prescribed stimulants, 6 were prescribed nonstimulants (1 was prescribed both), and two took a 24-hour medication hiatus prior to the MRI session, three did not, and seven did not indicate whether they took a hiatus.

Independent samples Mann-Whitney U tests indicated at-risk and not at-risk groups differed on IA, H/I, and ODD scores (all *p*_FDRcorr_<0.001) with those at-risk scoring higher on these measures. Groups did not differ on age, SES, perceptual or verbal reasoning, anxiety, or depression scores (all *p*_FDRcorr_>0.084).

### Normative Modeling Results

We initially used global measures of brain morphometry to investigate whether cortical thickness or subcortical gray matter volume are associated with ADHD at-risk status. Global models included the normative volume of total subcortical gray matter, the normative mean cortical thickness (separately for the two hemispheres), total ICV, motion covariates, anxiety and depression scores. The model including ODD scores predicted ADHD at-risk status (AUC = 0.879, *p* = .001), with a negative association of total subcortical gray matter volume (coef = -0.742, *p* = .009) and a positive association of ODD scores (coef = 1.63, *p* = .001) with ADHD at-risk status (Figure S1). The effect of non-interest of CSF SNR was also significant (coef = -0.806, *p* = .001). Despite not reaching a significant model fit (AUC = 0.71, *p* = .057), the model without ODD scores predicted ADHD at-risk status, with a negative association of total subcortical gray matter volume (coef = -0.306, *p* = .013) with ADHD at-risk status. The effects of non-interest of CSF SNR (coef = -0.364, *p* = .011) and GM SNRd (coef = -0.149, *p* = .039) were also significant. Cortical thickness was not associated with ADHD at-risk status in either model (all *p*s > 0.215).

**Fig. 1:**
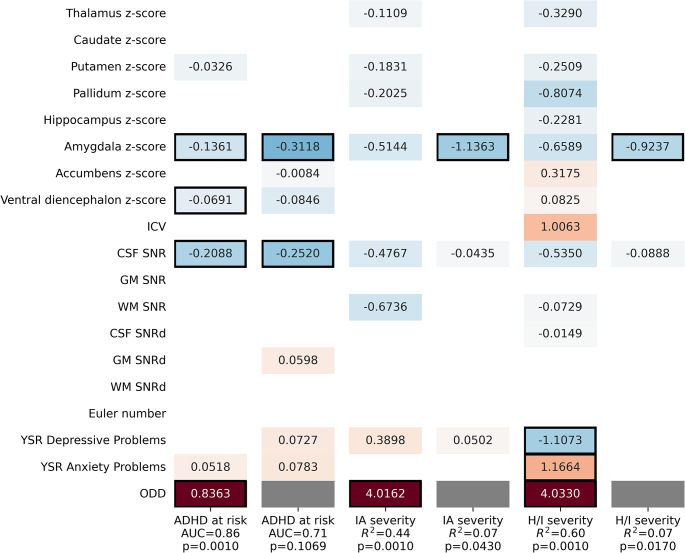
Modeling ADHD at risk status, IA and H/I severity using normative volumes of subcortical ROIs. Cells with black frames indicate significant (*p* < .05) coefficients and white cells indicate zero coefficients. Gray cells mark where the ODD predictor variable was omitted from the model

To further investigate the effect of subcortical gray matter volume on ADHD at-risk status, we defined subcortical models with the normative volume of subcortical ROIs as predictors, along with ICV, motion covariates, anxiety, and depression scores. In estimating ADHD at-risk status, the model including ODD scores predicted ADHD at-risk status (AUC = 0.858, *p* = .001), with a negative association of bilateral amygdala volume (coef = -0.136, *p* = .017), a negative association of bilateral ventral diencephalon (coef = -0.069, *p* = .047) and a positive association of ODD scores (coef = 0.836, *p* = .001) with ADHD at-risk status (Fig. 1). The effect of non-interest of CSF SNR was also significant (coef = -0.209, *p* = .009). Despite not reaching a significant model fit (AUC = 0.714, *p* = .107), the model without ODD scores predicted ADHD at-risk status, with a negative association of bilateral amygdala volume (coef = -0.312, *p* = .007) with ADHD at-risk status. The effect of non-interest of CSF SNR was also significant (coef = -0.252, *p* = .011).

In estimating severity of symptoms, models with ODD scores predicted IA (*R*^2^ = 0.442, *p* = .001) and H/I (*R*^2^ = 0.601, *p* = .001), with an association of ODD with IA (coef = 4.016, *p* = .001) and H/I (coef = 4.033, *p* = .001) and also of anxiety (coef = 1.166, *p* = .037) and depression (coef = -1.107, *p* = .033) scores with H/I. The models without ODD also predicted IA (*R*^2^ = 0.069, *p* = .043) and H/I (*R*^2^ = 0.073, *p* = .017), with a negative association of bilateral amygdala volume with IA (coef = -1.136, *p* = .003) and H/I (coef = -0.924, *p* = .001).

The inclusion of subcortical volumes in the ADHD risk model did not increase model fit (AUC) with ODD but did increase model fit without ODD by 0.06; in the IA severity model increased model fit (*R*^2^) by 0.01 with ODD and by 0.07 without ODD; and in the H/I severity model increased model fit (*R*^*2*^) by 0.06 with ODD and by 0.07 without ODD (see Figure S2 for the baseline model).

Normative modeling results were essentially replicated when omitting adolescents at-risk for ADHD who took ADHD medication (*n* = 12) and who took ADHD medication or another psychotropic medication (*n* = 14) (see Figure S4 and S5 for detailed results).

### Raw Modeling Results

**Fig. 2:**
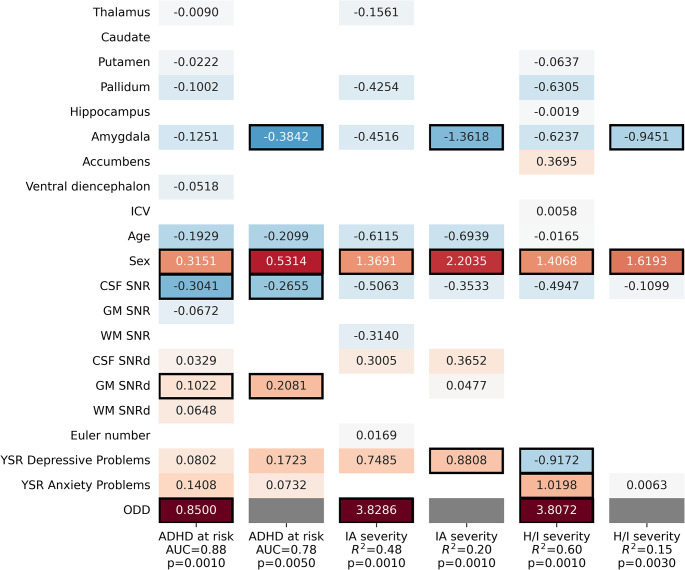
Modeling ADHD at risk status, IA and H/I severity using raw volumes of subcortical ROIs. Cells with black frames indicate significant (*p* < .05) coefficients and white cells indicate zero coefficients. Gray cells mark where the ODD predictor variable was omitted from the model

Subcortical models were recreated using the raw volumes of subcortical ROIs to assess whether and how findings obtained using normative modeling differ from those obtained not applying reference standards. In estimating ADHD at-risk status, the model including ODD scores predicted ADHD at-risk status (AUC = 0.876, *p* = .001), with a positive association of sex (boys scored higher; coef = 0.315, *p* = .007) and of ODD scores (coef = 0.850, *p* = .001) with ADHD at-risk status (Fig. 2). The effects of non-interest of CSF SNR (coef = -0.304, *p* = .013) and GM SNRd (coef = 0.102, *p* = .043) were also significant. The model without ODD scores predicted ADHD at-risk status (AUC = 0.776, *p* = .005) with a negative association of bilateral amygdala volume (coef = -0.384, *p* = .007) and a positive association of sex (boys scored higher; coef = 0.531, *p* = .003) with ADHD at-risk status. The effects of non-interest of CSF SNR (coef = -0.266, *p* = .023) and GM SNRd (coef = 0.208, *p* = .013) were also significant.

In estimating severity of symptoms, models with ODD scores predicted IA (*R*^2^ = 0.478, *p* = .001) and H/I (*R*^2^ = 0.597, *p* = .001), with an association of ODD with IA (coef = 3.827, *p* = .001) and H/I (coef = 3.807, *p* = .001); of sex with IA (boys scored higher; coef = 1.369, *p* = .005) and H/I (coef = 1.407, *p* = .013); and of anxiety (coef = 1.020, *p* = .047) and depression (coef = -0.917, *p* = .037) scores with H/I. The models without ODD predicted IA (*R*^2^ = 0.204, *p* = .001) and H/I (*R*^2^ = 0.148, *p* = .003), with a negative association of bilateral amygdala volume with IA (coef = -1.362, *p* = .001) and H/I (coef = -0.945, *p* = .003); a positive association of sex with IA (boys scored higher; coef = 2.204, *p* = .001) and H/I (coef = 1.619, *p* = .001); and a positive association of depression with IA (coef = 0.881, *p* = .021).

The inclusion of subcortical volumes in the ADHD risk model did not increase model fit (AUC) with ODD but did increase model fit without ODD by 0.04; in the IA severity model with ODD, fit (R^2^) was not increased but in the IA severity model without ODD, fit was increased by 0.05; and in the H/I severity model with ODD, fit (R^2^) was increased by 0.04 and in the H/I severity model without ODD, fit was increased by 0.08 (see Figure S3 for the baseline model).

## Discussion

Data in the current study indicate that relative to age- and sex-adjusted brain development norms, atypical amygdalar volume is associated with ADHD risk and severity in adolescents. Specifically, above and beyond the effects of the symptoms of common comorbidities including anxiety, depression and ODD as well as motion, reduced bilateral amygdala volume is associated with ADHD at-risk status and, above the effects of the symptoms of anxiety and depression as well as motion, reduced bilateral amygdala volume is associated with inattention and hyperactivity/impulsivity severity. These findings have conceptual and methodological implications.

Findings involving the amygdala are consistent with recent results of a cross-sectional mega-analysis showing smaller amygdala volumes in individuals with ADHD relative to without ADHD (Hoogman et al., 2017) and specifically suggest that above and beyond anxiety, depression, and ODD severity, individual differences in amygdala volume predict ADHD risk. Although the current study was not designed to address the functional significance of the obtained structural findings, it is noteworthy that the amygdala has been associated both with fear conditioning, and processing of negative and positive emotions (Baxter & Murray, 2002) as well as with reinforcement sensitivity (Baxter & Murray, 2002; Haber & Knutson, 2010; McClure et al., 2004; Zald, 2003). ADHD is also associated with greater negative (Healey et al., 2011; Martel & Nigg, 2006; Nigg et al., 2002; Singh & Waldman, 2010; White, 1999) and positive (Bunford et al., 2021; Forslund et al., 2016) affectivity and with atypical reinforcement sensitivity (Bunford et al., 2023; Hámori et al., 2023).

Of note, that the current findings were observed even when accounting for the effects of comorbidities underscores the utility of biological measures for predicting clinical outcome above and beyond more cost-effective alternatives (e.g., clinical and demographic measures) (Ball et al., 2014). Although inclusion of subcortical volumes systematically increased model fit, as expected, additional explained variance by subcortical volumes was relatively small. Data from large samples (which arguably generate more reliable/ less variable effect sizes) indicates that the association between biological measures and psychopathology indices is likely quite modest (Bunford et al., 2021; Kujawa & Burkhouse, 2017; Yancey et al., 2016), underscoring the clinical utility of constructing multi-method models, as we have done here, to improve estimation of outcomes and understanding of pathways across levels of analysis. Further, we also accounted for the effects of motion and ADHD and non-ADHD pharmacotherapy. Regarding accounting for the effects of motion, as expected, SNR-derived metrics, especially SNR of cerebrospinal fluid, were consistent predictors of ADHD, indicating greater motion-related artifacts in adolescents at risk for ADHD. Regarding accounting for the effects of ADHD and non-ADHD pharmacotherapy, although effect sizes were weaker in models without adolescents prescribed ADHD and non-ADHD pharmacotherapy, the main trends observed in primary analyses were replicated in these reanalyses, indicating the observed effects are neither driven nor distorted by pharmacological treatment.

Methodologically, these results show that recently published brain chart reference standards can be applied to address, in specific populations, clinically informative, focused and specific questions. Following others (Rutherford et al., 2023), we compared findings obtained using normative modeling to those obtained using raw data and essentially, results were replicated. Of note, amygdala effects in presence of ODD were only supported when using normative modeling, indicating a possible advantage of this novel approach relative to traditional approaches in identifying biological markers.

### Directions for Future Research

Our aim was to examine whether the normative modeling approach performs as well (comparably well) as a standard pipeline, in a relatively simple model (association between brain volume and ADHD risk and severity). As normative modeling of brain morphology is a rather novel approach, its application, especially when fitting it to an entirely new dataset is nontrivial. We believe that demonstrating that normative modeling can perform at least as well as raw modeling is an important first step in this line of research, before applying normative modeling to address more complex questions (e.g., association with functioning, prognosis, response to treatment).

We examined whether individual differences in brain volume predict ADHD risk status and severity. Although there is considerable utility in focusing on subclinical manifestations of the disorder, given emerging evidence that subclinical levels are associated with functional, negative outcomes (Rádosi et al., 2023) and may better capture such associations in girls whose ADHD is often more subtle (Takács et al., 2023), it will be important to determine whether current findings replicate across different classification thresholds, e.g., in predicting ADHD diagnostic status.

When ODD was included in the models as a covariate, amygdala volume was generally not significantly associated with ADHD risk or symptoms (except for one normative model with all participants). Conversely, when the effects of ODD were free to vary (i.e., it was not included as a covariate), amygdala volume was strongly associated with ADHD. The extent to which the herein observed pattern of results is driven in part by comorbid ODD symptomatology will also be necessary to uncover, e.g. in direct assessment of moderation.

In the current study, we applied regularized logistic and linear regression to uncover the effects of subcortical ROI volumes on ADHD risk status. Although predictive modeling of ADHD risk status with an independent test set or using nested cross validation could provide further insight into the underlying relationships in our data and potentially provide a tool for predicting ADHD risk status from new structural MRI scans, our dataset was deemed too small for such analyses. Nevertheless, this could be an important next step for our research.

Related, in the current study, we assessed the effects of the total cranial volume and of individual subcortical regional volume. Our dataset was too small to assess the effects of cortical regional volumes in the data-driven fashion that we employed in this study without making the estimation of effects unstable. This is another relevant next step for future research.

### Electronic Supplementary Material

Below is the link to the electronic supplementary material.

Supplementary Material 1

## References

[CR1] Achenbach, T. M., & Rescorla, L. A. (2001). *Manual for the ASEBA School-Age forms & profiles*. University of Vermont, Research Center for Children, Youth, & Families.

[CR2] Alexander-Bloch A, Clasen L, Stockman M, Ronan L, Lalonde F, Giedd J, Raznahan A (2016). Subtle in-scanner motion biases automated measurement of brain anatomy from in vivo MRI. Human Brain Mapping.

[CR3] American Psychiatric Association. (2022). *Diagnostic and Statistical Manual of Mental Disorders, Fifth Edition, text revision (DSM-5-TR)*. American Psychiatric Association.

[CR4] Ball TMM, Stein MBB, Paulus MPP (2014). Toward the application of functional neuroimaging to individualized treatment for anxiety and depression. Depression and Anxiety.

[CR5] Barkley RA (1997). Behavioral inhibition, sustained attention, and executive functions: Constructing a unifying theory of ADHD. Psychological Bulletin.

[CR6] Baxter, M. G., & Murray, E. A. (2002). The amygdala and reward. *Nature Reviews Neuroscience*, *3*(7). 10.1038/nrn875. Article 7. 10.1038/nrn875 12094212

[CR7] Bethlehem, R. I., Seidlitz, J., White, S. R., Vogel, J. W., Anderson, K. M., Adamson, C., Adler, S., Alexopoulos, G. S., Anagnostou, E., Areces-Gonzalez, A., Astle, D. E., Auyeung, B., Ayub, M., Bae, J., Ball, G., Baron-Cohen, S., Beare, R., Bedford, S. A., Benegal, V., & Alexander-Bloch, A. F. (2022). Brain charts for the human lifespan. *Nature*, *604*(7906). 10.1038/s41586-022-04554-y. Article 7906. 10.1038/s41586-022-04554-y PMC9021021 35388223

[CR8] Braver TS, Cole MW, Yarkoni T (2010). Vive les differences! Individual variation in neural mechanisms of executive control. Current Opinion in Neurobiology.

[CR10] Bunford N, Kujawa A, Dyson M, Olino T, Klein DN (2021). Developmental pathways from preschool temperament to early adolescent ADHD symptoms through initial responsiveness to reward. Development and Psychopathology.

[CR9] Bunford N, Hámori G, Nemoda Z, Angyal N, Fiáth R, Sebők-Welker TÉ, Pászthy B, Ulbert I, Réthelyi JM (2023). The domain-variant indirect association between electrophysiological response to reward and ADHD presentations is moderated by dopaminergic polymorphisms. Comprehensive Psychiatry.

[CR11] Buss DM (1991). Evolutionary personality psychology. Annual Review of Psychology.

[CR14] Castellanos FX, Tannock R (2002). Neuroscience of attention-deficit/hyperactivity disorder: The search for endophenotypes. Nature Reviews Neuroscience.

[CR12] Castellanos FX, Lee PP, Sharp W, Jeffries NO, Greenstein DK, Clasen LS, Blumenthal JD, James RS, Ebens CL, Walter JM, Zijdenbos A, Evans AC, Giedd JN, Rapoport JL (2002). Developmental trajectories of brain volume abnormalities in children and adolescents with attention-deficit/hyperactivity disorder. Journal of the American Medical Association.

[CR13] Castellanos FX, Sonuga-Barke EJS, Milham MP, Tannock R (2006). Characterizing cognition in ADHD: Beyond executive dysfunction. Trends in Cognitive Sciences.

[CR15] D’Agati E, Curatolo P, Mazzone L (2019). Comorbidity between ADHD and anxiety disorders across the lifespan. International Journal of Psychiatry in Clinical Practice.

[CR16] Danielson ML, Bitsko RH, Ghandour RM, Holbrook JR, Kogan MD, Blumberg SJ (2018). Prevalence of parent-reported ADHD diagnosis and Associated Treatment among U.S. children and adolescents, 2016. Journal of Clinical Child and Adolescent Psychology.

[CR17] DuPaul, G. J., Power, T. J., Anastopoulos, A. D., & Reid, R. (2016). *ADHD rating Scale-5 for children and adolescents*. The Guilford.

[CR18] Ellison-Wright I, Ellison-Wright Z, Bullmore E (2008). Structural brain change in attention deficit hyperactivity disorder identified by meta-analysis. Bmc Psychiatry.

[CR211] Esteban, O., Birman, D., Schaer, M., Koyejo, O. O., Poldrack, R. A., Gorgolewski, K. J. (2017). MRIQC: Advancing the automatic prediction of image quality in MRI from unseen sites. *PLOS ONE 12*, e0184661 . 10.1371/journal.pone.0184661 10.1371/journal.pone.0184661 PMC5612458 28945803

[CR19] Fair, D. A., Bathula, D., Nikolas, M. A., Nigg, J. T., Iyer, S., Bathula, D., Mills, K. L., Dosenbach, N. U. F., Schlaggar, B. L., Mennes, M., Gutman, D., Bangaru, S., Buitelaar, J. K., Dickstein, D. P., Di Martino, A., Kennedy, D. N., Kelly, C., Luna, B., Schweitzer, J. B., & Milham, M. P. (2012). Distinct neuropsychological subgroups in typically developing youth inform heterogeneity in children with ADHD. *Proceedings of the National Academy of Sciences*, *6*, 80. 10.1073/pnas.1115365109 10.1073/pnas.1115365109 PMC3340031 22474392

[CR20] Faraone SV, Biederman J, Mick E (2006). The age-dependent decline of attention deficit hyperactivity disorder: A meta-analysis of follow-up studies. Psychological Medicine.

[CR21] First, M. B., Williams, J. B. W., Karg, R. S., & Spitzer, R. L. (2016). In X. Gonda (Ed.), *SCID-5-CV (Klinikai változat). Strukturált Klinikai interjú a DSM-5® zavarok felmérésére*. Oriold és Társai Ltd.

[CR210] Fischl, B., Salat, D. H., Busa, E., Albert, M., Dieterich, M., Haselgrove, C., Kouwe, A. van der, Killiany, R., Kennedy, D., Klaveness, S., Montillo, A., Makris, N., Rosen, B., Dale, A. M. (2002). Whole brain segmentation: Automated labeling of neuroanatomical structures in the human brain. *Neuron*, *33*, 341–355. 10.1016/S0896-6273(02)00569-X 10.1016/s0896-6273(02)00569-x 11832223

[CR22] Forslund T, Brocki KC, Bohlin G, Granqvist P, Eninger L (2016). The heterogeneity of attention-deficit/hyperactivity disorder symptoms and conduct problems: Cognitive inhibition, emotion regulation, emotionality, and disorganized attachment. British Journal of Developmental Psychology.

[CR23] Frodl T, Skokauskas N (2012). Meta-analysis of structural MRI studies in children and adults with attention deficit hyperactivity disorder indicates treatment effects. Acta Psychiatrica Scandinavica.

[CR24] Gilmore AD, Buser NJ, Hanson JL (2021). Variations in structural MRI quality significantly impact commonly used measures of brain anatomy. Brain Informatics.

[CR25] Gnanavel S, Sharma P, Kaushal P, Hussain S (2019). Attention deficit hyperactivity disorder and comorbidity: A review of literature. World Journal of Clinical Cases.

[CR26] Haber, S. N., & Knutson, B. (2010). The reward circuit: Linking primate anatomy and human imaging. *Neuropsychopharmacology : Official Publication of the American College of Neuropsychopharmacology*, *35*(1). 10.1038/npp.2009.129 10.1038/npp.2009.129 PMC3055449 19812543

[CR27] Halperin JM, Schulz KP (2006). Revisiting the role of the prefrontal cortex in the pathophysiology of attention-deficit/hyperactivity disorder. Psychological Bulletin.

[CR28] Hámori G, File B, Fiáth R, Pászthy B, Réthelyi JM, Ulbert I, Bunford N (2023). Adolescent ADHD and electrophysiological reward responsiveness: A machine learning approach to evaluate classification accuracy and prognosis. Psychiatry Research.

[CR29] Healey DM, Marks DJ, Halperin JM (2011). Examining the interplay among negative emotionality, cognitive functioning, and attention deficit/hyperactivity disorder symptom severity. Journal of the International Neuropsychological Society.

[CR31] Hoogman M, Rijpkema M, Janss L, Brunner H, Fernandez G, Buitelaar J, Franke B, Arias-Vásquez A (2012). Current self-reported symptoms of attention deficit/hyperactivity disorder are associated with total brain volume in healthy adults. PloS One.

[CR30] Hoogman M, Bralten J, Hibar DP, Mennes M, Zwiers MP, Schweren L, van Hulzen KJE, Medland SE, Shumskaya E, Jahanshad N, de Zeeuw P, Szekely E, Sudre G, Wolfers T, Onnink AMH, Dammers JT, Mostert JC, Vives-Gilabert Y, Kohls G, Franke B (2017). Subcortical brain volume differences of participants with ADHD across the lifespan: An ENIGMA collaboration. The Lancet Psychiatry.

[CR32] Hungarian Central Statistical Office. (n.d.). *A háztartások életszínvonala, 2020*. Retrieved September 14 (2023). from https://www.ksh.hu/docs/hun/xftp/idoszaki/hazteletszinv/2020/index.html

[CR33] Kujawa A, Burkhouse KL (2017). Vulnerability to Depression in Youth: Advances from affective neuroscience. Biological Psychiatry: Cognitive Neuroscience and Neuroimaging.

[CR34] Martel MM, Nigg JT (2006). Child ADHD and personality/temperament traits of reactive and effortful control, resiliency, and emotionality. Journal of Child Psychology and Psychiatry and Allied Disciplines.

[CR35] McClure SM, York MK, Montague PR (2004). The neural substrates of reward processing in humans: The modern role of FMRI. The Neuroscientist: A Review Journal Bringing Neurobiology Neurology and Psychiatry.

[CR36] *MRIQC: Advancing the automatic prediction of image quality in MRI from unseen sites | PLOS ONE*. (n.d.). Retrieved August 2 (2023). from https://journals.plos.org/plosone/article?id=10.1371/journal.pone.0184661 10.1371/journal.pone.0184661 PMC5612458 28945803

[CR37] Nakao T, Radua J, Rubia K, Mataix-Cols D (2011). Gray matter volume abnormalities in ADHD: Voxel-based meta-analysis exploring the effects of age and stimulant medication. The American Journal of Psychiatry.

[CR38] Nárai, Á., Hermann, P., Auer, T., Kemenczky, P., Szalma, J., Homolya, I., Somogyi, E., Vakli, P., Weiss, B., & Vidnyánszky, Z. (2022). Movement-related artefacts (MR-ART) dataset of matched motion-corrupted and clean structural MRI brain scans. *Scientific Data*, *9*(1). 10.1038/s41597-022-01694-8 10.1038/s41597-022-01694-8 PMC9576686 36253426

[CR39] Nigg JT, John OP, Blaskey LG, Huang-Pollock CL, Willcutt EG, Hinshaw SP, Pennington B, John OP, Willcutt EG, Pennington B, Blaskey LG, Huang-Pollock CL, Willcutt EG, Hinshaw SP, Pennington B (2002). Big five dimensions and ADHD symptoms: Links between personality traits and clinical symptoms. Journal of Personality and Social Psychology.

[CR40] Norman LJ, Carlisi C, Lukito S, Hart H, Mataix-Cols D, Radua J, Rubia K (2016). Structural and functional brain abnormalities in Attention-Deficit/Hyperactivity disorder and obsessive-compulsive disorder: A comparative Meta-analysis. JAMA Psychiatry.

[CR41] Pardoe HR, Hiess K, Kuzniecky R (2016). Motion and morphometry in clinical and nonclinical populations. Neuroimage.

[CR42] Pedregosa F, Varoquaux G, Gramfort A, Michel V, Thirion B, Grisel O, Blondel M, Prettenhofer P, Weiss R, Dubourg V, Vanderplas J, Passos A, Cournapeau D, Brucher M, Perrot M, Duchesnay É (2011). Scikit-learn: Machine learning in Python. Journal of Machine Learning Research.

[CR43] Pillow DR, Pelham WE, Hoza B, Molina BSG, Stultz CH (1998). Confirmatory factor analyses examining attention deficit hyperactivity disorder symptoms and other childhood disruptive behaviors. Journal of Abnormal Child Psychology.

[CR44] Provins C, MacNicol E, Savary E, Hagmann P, Esteban O (2023). Assessment of B1 field dynamics of rats BOLD fMRI using the wavelet transform. OSF Preprints.

[CR45] Rádosi A, Ágrez K, Pászthy B, Réthelyi JM, Ulbert I, Bunford N (2023). Concurrent and prospective associations of reward response with affective and alcohol problems: ADHD-Related Differential Vulnerability. Journal of Youth and Adolescence.

[CR46] Reuter M, Tisdall MD, Qureshi A, Buckner RL, van der Kouwe AJ, Fischl B (2015). Head Motion during MRI Acquisition reduces Gray Matter volume and thickness estimates. Neuroimage.

[CR47] Rosen AFG, Roalf DR, Ruparel K, Blake J, Seelaus K, Villa LP, Ciric R, Cook PA, Davatzikos C, Elliott MA, Garcia de La Garza A, Gennatas ED, Quarmley M, Schmitt JE, Shinohara RT, Tisdall MD, Craddock RC, Gur RE, Gur RC, Satterthwaite TD (2018). Quantitative assessment of structural image quality. Neuroimage.

[CR48] Roy A, Hechtman L, Roy A, Arnold LE, Sibley MH, Molina BSG, Swanson JM, Howard AL, Vitiello B, Severe JB, Jensen PS, Arnold LE, Hoagwood K, Richters J, Vereen D, Hinshaw SP, Elliott GR, Wells KC, Epstein JN, Stern K (2016). Childhood factors affecting persistence and desistence of Attention-Deficit/Hyperactivity disorder symptoms in Adulthood: Results from the MTA. Journal of the American Academy of Child and Adolescent Psychiatry.

[CR50] Rutherford S, Fraza C, Dinga R, Kia SM, Wolfers T, Zabihi M, Berthet P, Worker A, Verdi S, Andrews D, Han LK, Bayer JM, Dazzan P, McGuire P, Mocking RT, Schene A, Sripada C, Tso IF, Duval ER, Marquand AF (2022). Charting brain growth and aging at high spatial precision. eLife.

[CR49] Rutherford S, Barkema P, Tso IF, Sripada C, Beckmann CF, Ruhe HG, Marquand AF (2023). Evidence for embracing normative modeling. eLife.

[CR51] Salari N, Ghasemi H, Abdoli N, Rahmani A, Shiri MH, Hashemian AH, Akbari H, Mohammadi M (2023). The global prevalence of ADHD in children and adolescents: A systematic review and meta-analysis. Italian Journal of Pediatrics.

[CR52] Sciberras E, Streatfeild J, Ceccato T, Pezzullo L, Scott J, Middeldorp C, Hutchins, Paterson R, Bellgrove M, Coghill D (2022). Social and economic costs of Attention-Deficit/Hyperactivity disorder across the Lifespan. Journal of Attention Disorders.

[CR53] Shaw P, Gilliam M, Liverpool M, Weddle C, Malek M, Sharp W, Greenstein D, Evans A, Rapoport J, Giedd J (2011). Cortical development in typically developing children with symptoms of hyperactivity and impulsivity: Support for a dimensional view of attention deficit hyperactivity disorder. The American Journal of Psychiatry.

[CR54] Singh AL, Waldman ID (2010). The etiology of associations between negative emotionality and childhood externalizing disorders. Journal of Abnormal Psychology.

[CR55] Sonuga-Barke EJS (2003). The dual pathway model of AD/HD: An elaboration of neuro-developmental characteristics. Neuroscience & Biobehavioral Reviews.

[CR56] Sonuga-Barke EJS, Bitsakou P, Thompson M (2010). Beyond the dual pathway model: Evidence for the dissociation of timing, Inhibitory, and Delay-related impairments in Attention-Deficit/Hyperactivity disorder. Journal of the American Academy of Child and Adolescent Psychiatry.

[CR57] Stoet G (2010). PsyToolkit: A software package for programming psychological experiments using Linux. Behavior Research Methods.

[CR58] Stoet G (2017). PsyToolkit: A novel web-based method for running online questionnaires and reaction-time experiments. Teaching of Psychology.

[CR59] Takács, M., Tóth, B., Szalárdy, O., & Bunford, N. (2023). Theta and alpha activity are differentially associated with physiological and rating scale measures of affective processing in adolescents with but not without ADHD. *Development and Psychopathology*, 1–16. 10.1017/S0954579423000639 10.1017/S0954579423000639 37357942

[CR60] Thomas R, Sanders S, Doust J, Beller E, Glasziou P (2015). Prevalence of Attention-Deficit/Hyperactivity disorder: A systematic review and Meta-analysis. Pediatrics.

[CR61] Valera EM, Faraone SV, Murray KE, Seidman LJ (2007). Meta-analysis of structural imaging findings in attention-deficit/hyperactivity disorder. Biological Psychiatry.

[CR62] Van Eck K, Finney SJ, Evans SW (2010). Parent report of ADHD symptoms of early adolescents: A confirmatory factor analysis of the disruptive Behavior disorders Scale. Educational & Psychological Measurement.

[CR63] Wechsler, D. (2003). *Wechsler intelligence scale for children–Fourth Edition (WISC-IV)*. The Psychological Corporation.

[CR64] Wechsler, D. (2008). *Wechsler adult intelligence scale–Fourth Edition (WAIS–IV)*. APA PsycTests.

[CR65] White JD (1999). Personality, temperament and ADHD: A review of the literature. Personality and Individual Differences.

[CR66] *Whole brain segmentation: Automated labeling of neuroanatomical structures in the human brain—PubMed*. (n.d.). Retrieved August 2, (2023). from https://pubmed.ncbi.nlm.nih.gov/11832223/10.1016/s0896-6273(02)00569-x 11832223

[CR67] Yancey JR, Venables NC, Patrick CJ (2016). Psychoneurometric operationalization of threat sensitivity: Relations with clinical symptom and physiological response criteria. Psychophysiology.

[CR68] Zald DH (2003). The human amygdala and the emotional evaluation of sensory stimuli. Brain Research Brain Research Reviews.

