# Supplementary material for: Amygdala Volume is Associated with ADHD Risk and Severity Beyond Comorbidities in Adolescents: Clinical Testing of Brain Chart Reference Standards
Source: Res Child Adolesc Psychopathol. 2024 Mar 14;52(7):1063–74. doi: 10.1007/s10802-024-01190-0 (PMC11217056; doi:10.1007/s10802-024-01190-0)
Supplement: Supplementary file 1 — Supplementary Material 1 [file 10802_2024_1190_MOESM1_ESM.docx]

SUPPLEMENTARY INFORMATION TO:

AMYGDALA VOLUME IS ASSOCIATED WITH ADHD RISK AND SEVERITY BEYOND COMORBIDITIES IN ADOLESCENTS:

CLINICAL TESTING OF BRAIN CHART REFERENCE STANDARDS


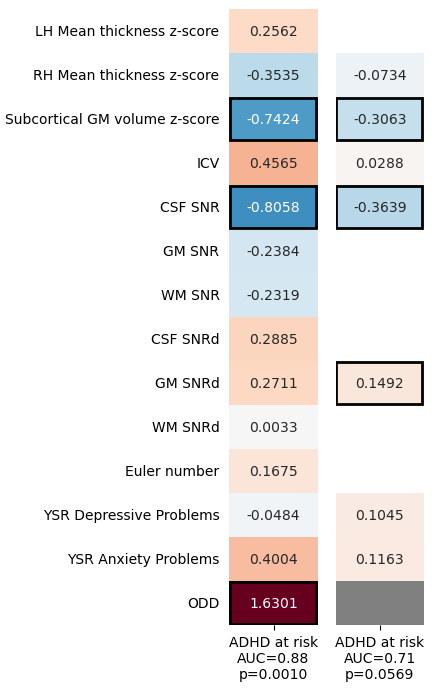


**Figure S1. Modeling ADHD at risk status using normative mean cortical thickness (separately for the two hemispheres) and subcortical gray matter (GM) volume.** Cells with black frames indicate significant (*p*<.05) coefficients and white cells indicate zero coefficients. Gray cells mark where the ODD predictor variable was omitted from the model.


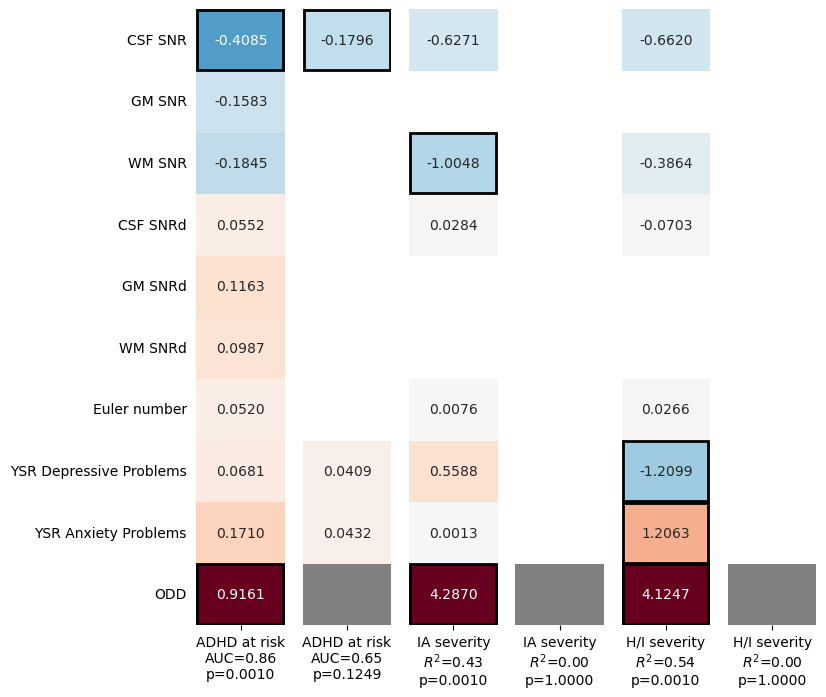


**Figure S2. Modeling ADHD at risk status, IA and H/I severity using only motion covariates and comorbidities.** Cells with black frames indicate significant (*p*<.05) coefficients and white cells indicate zero coefficients. Gray cells mark where the ODD predictor variable was omitted from the model.


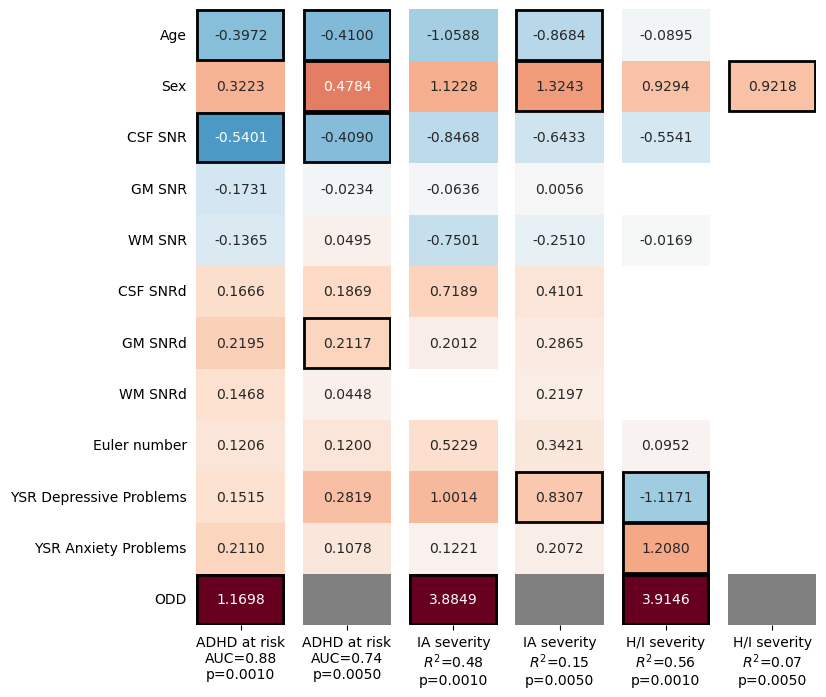


**Figure S3. Modeling ADHD at risk status, IA and H/I severity using only age, sex, motion covariates and comorbidities.** Cells with black frames indicate significant (*p*<.05) coefficients and white cells indicate zero coefficients. Gray cells mark where the ODD predictor variable was omitted from the model.

**
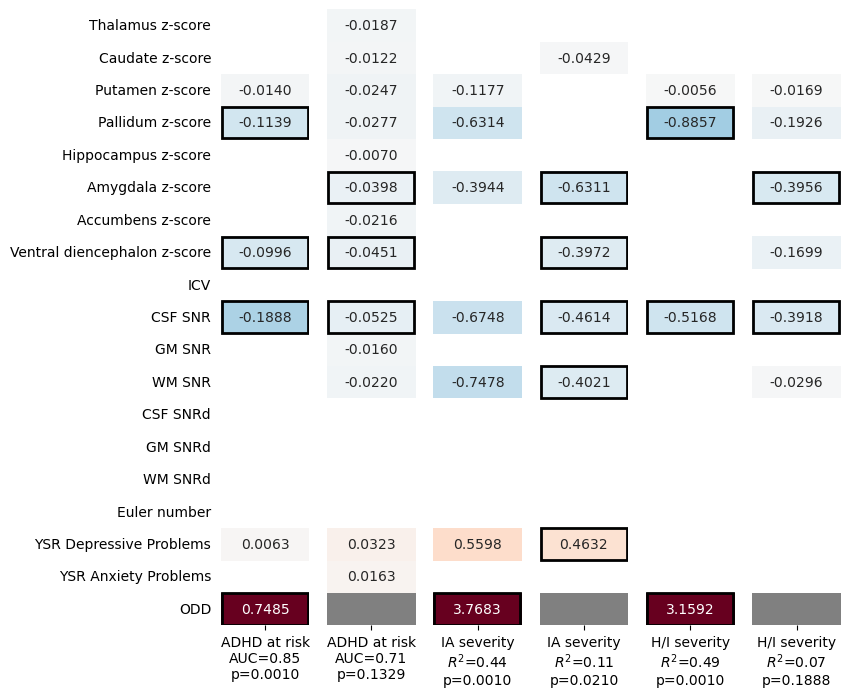
**

**Figure S4. Modeling ADHD at risk status, IA and H/I severity of participants not taking ADHD medication using normative volumes of subcortical ROIs.** Cells with black frames indicate significant (*p*<.05) coefficients and white cells indicate zero coefficients. Gray cells mark where the ODD predictor variable was omitted from the model.


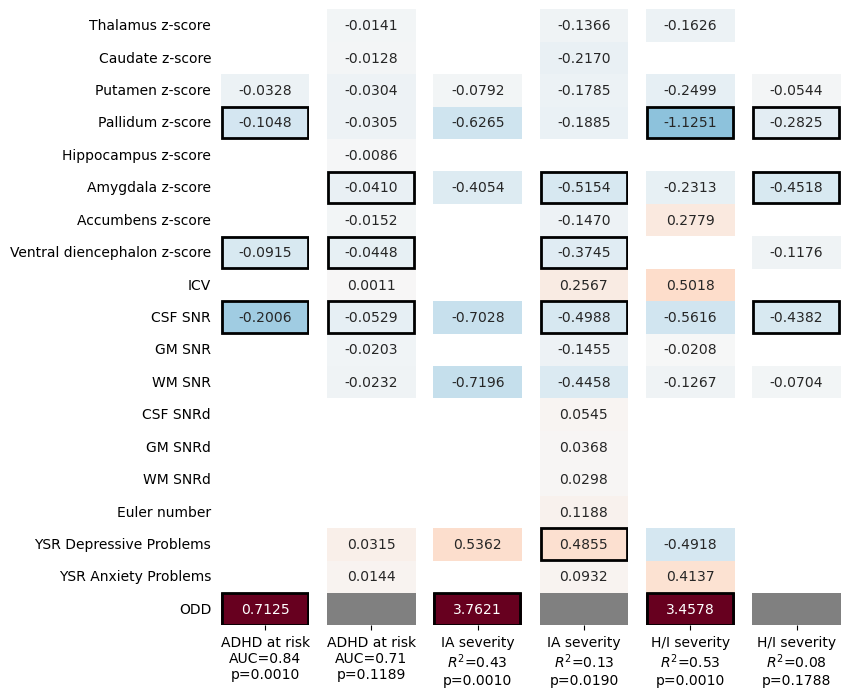


**Figure S5. Modeling ADHD at risk status, IA and H/I severity of participants not taking ADHD or other psychotropic medication using normative volumes of subcortical ROIs.** Cells with black frames indicate significant (*p*<.05) coefficients and white cells indicate zero coefficients. Gray cells mark where the ODD predictor variable was omitted from the model.
